# An unusual case of recurrent traumatic spinal epidural hematoma accompanied by osteoporotic vertebral compression fractures: Case report

**DOI:** 10.1097/MD.0000000000039650

**Published:** 2024-10-04

**Authors:** Yake Meng, Kai Su, Yaojun Dai, Xiaopan Chang, Yong Yang, Wei Mei, Wei Zhang, Hao Pan

**Affiliations:** a Department of Orthopedics, Hangzhou Hospital of Traditional Chinese Medicine, Zhejiang Chinese Traditional Medicine University, Zhejiang, China; b Department of Orthopedics, Zhengzhou Orthopedic Hospital, Henan University, Henan, China.

**Keywords:** osteoporotic vertebral compression fractures, spinal epidural hematoma, vertebroplasty

## Abstract

**Rationale:**

Traumatic spinal epidural hematoma (SEH) is a rare clinical condition. Here, we present an extraordinary case of recurrent SEH accompanied by thoracolumbar spine fractures resulting from minor trauma, and provide evidence-based recommendations for the surgical management strategies in this unique scenario.

**Patient concerns:**

A 71-year-old female patient presented with back pain after a fall. Magnetic resonance imaging revealed an SEH with L2 vertebral compression fracture. Following unsuccessful conservative treatment, percutaneous vertebroplasty was performed at the 2nd lumbar vertebra under local anesthesia. Two years later, the patient experienced another fall and was diagnosed with spinal hematoma with L1 vertebral compression fractures.

**Diagnoses:**

The patient was diagnosed with recurrent osteoporotic vertebral compression fracture accompanying SEH.

**Outcomes:**

After 1 week of conservative treatment, notable improvement of limbs numbness was observed. The patient ultimately underwent L1 vertebroplasty surgery. The patient was discharged smoothly on the third postoperative day and made a full recovery after 4 months.

**Lessons:**

SEH is a rare clinical finding that can occur even after a minor trauma in the elderly. It is worth noting that osteoporotic vertebral compression fractures with asymptomatic or stable intraspinal hematoma, is not considered to be a contraindication for percutaneous vertebroplasty. And percutaneous vertebroplasty is a safe and effective treatment for osteoporotic compression fractures with asymptomatic SEH.

## 1. Introduction

Spinal epidural hematoma (SEH) is a rare condition that can potentially cause significant neurological impairment. The etiology of SEH may involve various factors such as idiopathic origins, traumatic events, or iatrogenic interventions.^[1]^ Pathogenesis of SEH is not completely understood. And the epidural hematoma hypothesis states that internal posterior epidural venous plexus is the anatomic structure responsible for the hematoma. Traumatic SEH is a relatively uncommon injury that results from the mechanical disruption of blood vessels in the epidural space by vertebral fracture fragments or dislocation.^[2,3]^ In this report, we present an exceptional case involving recurrent episodes of SEH with thoracolumbar spine fractures following minor trauma. And, we aim to provide evidence-based recommendations for the surgical management strategies in this unique scenario.

## 2. Case presentation

**Figure 1.**
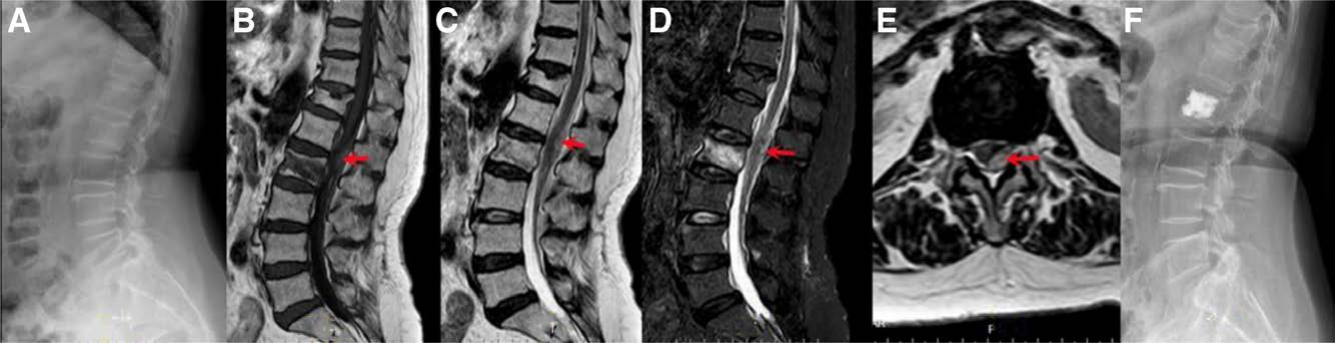
(A) X-rays showed vertebral compression fractures at T12 and L2. (B–E) MRI showed acute compression fracture of L2 body and epidural hematoma extending from L2 to L1. (F) X-rays showed that bone cement was well localized in the L2 vertebral body. MRI = magnetic resonance imaging.

A 71-year-old female patient presented with intense back pain after a fall at the emergency department. Physical examination revealed limited mobility of the lumbar spine in all planes, without apparent motor or sensory deficits. Her past medical history was unremarkable, with no prior use of anticoagulant medications. Blood tests, including white cell count, inflammatory markers, tumor markers, blood calcium, and blood phosphorus analysis, were all within normal limits. And her coagulogram showed no abnormalities. Radiographic examination of the thoracolumbar spine revealed osteoporotic compression fractures in the 12th thoracic vertebra and the second lumbar vertebra (Fig. 1A). Magnetic resonance imaging (MRI) of the thoracolumbar spine showed a recent benign compression fracture of the L2 body, an acute atypical hematoma extending from L2 to L1, and an old fracture of the T12 body (Fig. 1B–E). Bone mineral density (BMD) examination revealed severe osteoporosis, with a lumbar spine T-score of −3.1. Under local anesthesia, percutaneous vertebroplasty (PVP) was performed on the second lumbar vertebra (Fig. 1F). The patient experienced immediate relief of pain following the surgery with no postoperative complications. She was discharged smoothly on the second day after the operation. One month postoperatively, the patient became asymptomatic and returned to normal life. She received zoledronic acid injections and continued to be regularly monitored at our outpatient endocrinology clinic, necessitating ongoing osteoporosis drug therapy (salmon calcitonin nasal spray, calcium agents).

**Figure 2.**
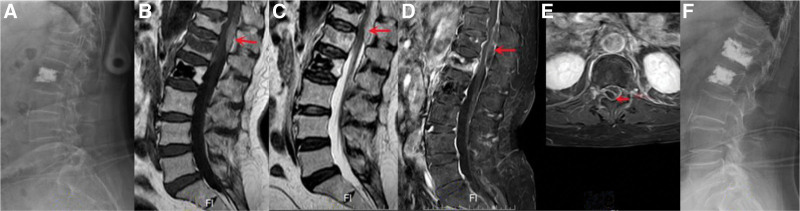
(A) X-rays showed vertebral compression fractures at T12 and L1, and a vertebroplasty of L2. (B, C) MRI showed acute compression fracture of L1 body and epidural hematoma extending from T12 to L1. (D, E) Enhanced MRI showed acute compression fracture of L1 body and epidural hematoma extending from T12 to L1 with annular enhancement. (F) X-rays showed that bone cement was well localized in the L1 vertebral body. MRI = magnetic resonance imaging.

Two years later, the patient returned to our clinic, and presented with severe low back pain and numbness in the lower extremities following a minor fall. Physical examination showed no apparent motor or sensory abnormalities. Blood tests, including white cell count, inflammatory markers, tumor markers, blood calcium, and blood phosphorus analysis, were within normal limits. Radiological assessment revealed compression fractures in the L1 vertebra, and preexisting compression fractures of the L2 and T12 vertebrae (Fig. 2A). BMD examination confirmed severe osteoporosis, with a lumbar spine T-score of −2.7. MRI revealed an acute compression fracture of the L1 body, and a dorsal epidural soft tissue mass that resulted in posterior compression of the spinal cord at the T11-L1 levels (Fig. 2B and C). A diagnosis of traumatic spinal cord hematoma with L1 vertebral compression fracture was made. Surgical decompression and hematoma evacuation were recommended; however, the patient refused to the full open operation. Following 3 days of conservative treatment, the patient reported significant improvement in limb numbness, with only residual severe back pain. Subsequently, an enhanced MRI was performed, revealed the presence of the epidural hematoma without any signs of hematoma enlargement (Fig. 2D and E). After 7 days of conservative treatment, the numbness in the lower limbs exhibited remarkable improvement, and the patient undergo PVP surgery (Fig. 2F). The back pain was markedly alleviated postoperatively, without any complications. The patient was discharged from the hospital smoothly on the third day after the operation and remained in good condition 4 months later, with no residual numbness in the lower extremities. The postoperative course was uneventful and the woman returned to a normal life.

## 3. Discussion and conclusions

The clinical case of spinal hematoma was first reported by Jackson.^[4]^ Subsequently, numerous case reports have been reported in the literature. While SEH can occur anywhere in the spinal cord, it predominantly occurs dorsally to the spinal cord in the cervicothoracic and thoracolumbar regions.^[1,2]^ This can be attributed to the fact that the dura mater adheres more closely to the posterior longitudinal ligament compared to the ligamenta flava.^[1,2]^

SEH is a relatively uncommon yet under-appreciated and potentially life-threatening neurological condition, and recurrent SEH is even more rare. Harik et al^[5]^ reported the first case of recurrent SEH in a patient receiving anticoagulant therapy. Luo et al^[6]^ classified the reported cases of recurrent SEH into 2 categories: spontaneous and postoperative. Traumatic SEH, on the other hand, is less frequently encountered. The incidence of spinal hematoma in patients with spinal trauma has been reported to range from 1% to 1.7%.^[1]^ And the prevalence of SEH associated with vertebral fractures has been reported to range from 0.5% to 7.5%.^[7]^ Risk factors for traumatic SEH include high-energy trauma, cervical spondylosis, rheumatoid arthritis, Paget disease, and ankylosing spondylitis.^[7]^ However, there are scarce reports of osteoporotic vertebral compression fracture (OVCF) accompanying SEH. To the best of our knowledge, our case should be the first reported case of recurrent traumatic SEH associated with OVCF in the world literature.

The spine MRI scans are strongly recommended for the elderly with traumatic back pain. MRI has high diagnostic sensitivity and accuracy for assessing new spinal fractures. Moreover, MRI images play a pivotal role in the prompt identification, precise diagnosis, and ongoing monitoring of SEH.^[8]^ The optimal approach to SEH treatment remains a subject of heated debate. Surgical intervention is considered the mainstay of treatment in patients with SEH experiencing neurological deficits.^[1,2]^ Early decompression surgery is associated with improved neurological recovery. However, conservative treatment was considered first in patients with minimal or no neurological deficits.^[1,2]^ Furthermore, a growing number of reports documenting rapid and spontaneous recovery in cases of SEH also supports the notion of conservative follow-up.^[9]^ The occurrence of SEH with OVCF resulting from minimal trauma is exceedingly rare. Oda et al^[10]^ reported a singular case of myelopathy caused by chronic SEH with L1 osteoporotic vertebral collapse, which was successfully managed through posterior decompression and fixation surgery. It has been established that epidural hematomas caused by spinal trauma typically resolve within a 3-week in most cases.^[9]^ Hirata et al^[2]^ and Anand et al^[11]^ reported a successful utilization of standalone vertebroplasty as a treatment approach for OVCF with SEH.^[2,11]^ In this study, we present a unique case of recurrent SEH with OVCF following low-energy trauma. The patient experienced 2 episodes of bleeding at a 2-year interval and subsequently underwent vertebroplasty surgery. Although there is no follow-up information regarding the absorption of the hematoma, the patient ultimately achieved satisfactory clinical outcomes. OVCF with asymptomatic or stable hematoma should not be considered a contraindication for PVP. And, PVP is considered a safe and effective option in the management of OVCF combined with stable spinal hematoma. However, the potential risk of exacerbation of spinal hematoma as a result of vertebroplasty warrants further investigation. Additionally, vertebroplasty is an invasive technique and although its complications are rare, they can be life-threatening, including cement leakage. Fortunately, many potential complications can be prevented or minimized through the standard therapeutic procedures.

Osteoporosis, or low BMD, is the single most important risk factor for osteoporotic spinal compression fractures, especially in older postmenopausal women. In our case, the patient with prevalent vertebral fractures and severe osteoporosis. Therefore, it is important to consider systemic anti-osteoporotic treatment as preventive and therapeutic measures among patients with osteoporosis. Moreover, active anti-osteoporosis treatment and various effective measures for preventing falls are vital for preventing relapse.

The downside of these studies is limitation in number of samples. And the underlying mechanism of OVCF accompanied by SHE is unclear, especially in the patients with recurrent attacks, thus, additional in-depth researches are needed.

SEH is a rare clinical finding that can occur even after a minor trauma in the elderly. To the best of our knowledge, this is the first documented case of recurrent SEH with thoracolumbar spine fractures resulting from minor trauma. It is imperative for clinicians to meticulously review the imaging data to avoid overlooking such a diagnosis. PVP is an effective treatment for OVCFs with stable spinal hematoma.

## Author contributions

**Data curation:** Yake Meng.

**Formal analysis:** Yake Meng, Hao Pan.

**Investigation:** Kai Su, Hao Pan.

**Resources:** Yaojun Dai.

**Writing – original draft:** Xiaopan Chang.

**Writing – review & editing:** Yong Yang.

**Software:** Wei Mei.

**Methodology:** Wei Zhang.
